# Autism spectrum disorder in children born preterm—role of exposure to perinatal inflammation

**DOI:** 10.3389/fnins.2013.00123

**Published:** 2013-07-22

**Authors:** Suzanne J. Meldrum, T. Strunk, A. Currie, S. L. Prescott, K. Simmer, A. J. O. Whitehouse

**Affiliations:** ^1^School of Paediatrics and Child Health, The University of Western AustraliaCrawley, Perth, WA, Australia; ^2^Centre for Neonatal Research and Education, University of Western AustraliaPerth, WA, Australia; ^3^School of Veterinary and Life Sciences, Murdoch UniversityMurdoch, WA, Australia; ^4^Telethon Institute for Child Health Research, University of Western AustraliaCrawley, WA, Australia; ^5^School of Psychology, University of Western AustraliaCrawley, WA, Australia

**Keywords:** preterm, autism spectrum disorders, prenatal infection, immunology

## Abstract

Autism Spectrum Disorder (ASD) is the collective term for neurodevelopmental disorders characterized by qualitative impairments in social interaction, communication, and a restricted range of activities and interests. Many countries, including Australia, have reported a dramatic increase in the number of diagnoses over the past three decades, with current prevalence of ASD at 1 in every 110 individuals (~1%). The potential role for an immune-mediated mechanism in ASD has been implicated by several studies, and some evidence suggests a potential link between prenatal infection-driven inflammation and subsequent development of ASD. Furthermore, a modest number of contemporary studies have reported a markedly increased prevalence of ASD in children born preterm, who are at highest risk of exposure to perinatal inflammation. However, the mechanisms that underpin the susceptibility to infection-driven inflammation during pregnancy and risk of preterm birth, and how these intersect with the subsequent development of ASD in the offspring, is not understood. This review aims to summarize and discuss the potential mechanisms and evidence for the role of prenatal infection on the central nervous system, and how it may increase the susceptibility for ASD pathogenesis in children born preterm.

## Introduction

Autism Spectrum Disorder (ASD) is the collective term for neurodevelopmental disorders characterized by qualitative impairments in social interaction, communication, and a restricted range of activities and interests (Association, 2000). Autistic Disorder, Pervasive Developmental Disorder—Not Otherwise Specified (PDD-NOS) and Asperger's Disorder differ with regard to the quality and quantity of symptoms, but are thought to share a similar genetic liability (Freitag, 2007). ASD is a growing public health concern. Many countries, including Australia (Williams et al., 2008b), have reported a dramatic increase in the number of ASD diagnoses over the past three decades from less than 1 in 1000 individuals with a form of ASD (Chakrabarti and Fombonne, 2001; Wing and Potter, 2002) to 1 in every 110 (~1%) (CDC, 2006). While some assessment factors may be responsible for part of the increase, including changes in diagnostic criteria, different assessment instruments and increased autism awareness (Matson and Kozlowski, 2011), several environmental and epigenetic factors have also been implicated. In particular, a range of perinatal exposures are associated with ASD, including heavy metal and pesticide exposure, stress, smoking, and use of anti-depressant medication during pregnancy and certain foods (Dietert et al., 2011). These same factors are also associated with preterm birth, and it is important to note that a number of contemporary studies have reported an increased prevalence of ASD in preterm populations (Kolevzon et al., 2007).

## Prematurity and ASD

A meta-analysis of seven retrospective epidemiologic studies, representing five different geographic locations concluded that the following factors were associated with an increased risk of ASD; low birth weight, reduced gestational age at birth, birth asphyxia, and advanced maternal age (>35 years), along with a maternal place of birth outside Europe/North America (Kolevzon et al., 2007). A further five epidemiological studies published after 2007 found similar associations (Schendel and Bhasin, 2008; Williams et al., 2008a; Buchmayer et al., 2009; Schieve et al., 2011; Guinchat et al., 2012; Lampi et al., 2012; Movsas and Paneth, 2012), with the exception of Schieve et al. (2011), who found that prematurity did not account for a significant proportion of the increase in ASD prevalence. Yet this study addressed the predictive factors associated with the recent increase in ASD prevalence, rather than ASD diagnosis per se. A caveat to these findings, however, there was an over-representation of children with severe forms of ASD, indicating that these studies may suffer from self-selection bias.

In terms of gestational age, there is evidence that the qualitative and quantitative nature of ASD symptomatology may differ across gestational age bands. Movsas and Paneth (2012) identified that for infants born less than 34 weeks, the largest impairments were within the domains of social cognition, social communication and autistic mannerisms. For children born between 34 and 36 weeks, all domains were equally affected (Movsas and Paneth, 2012).

**Figure 1:**
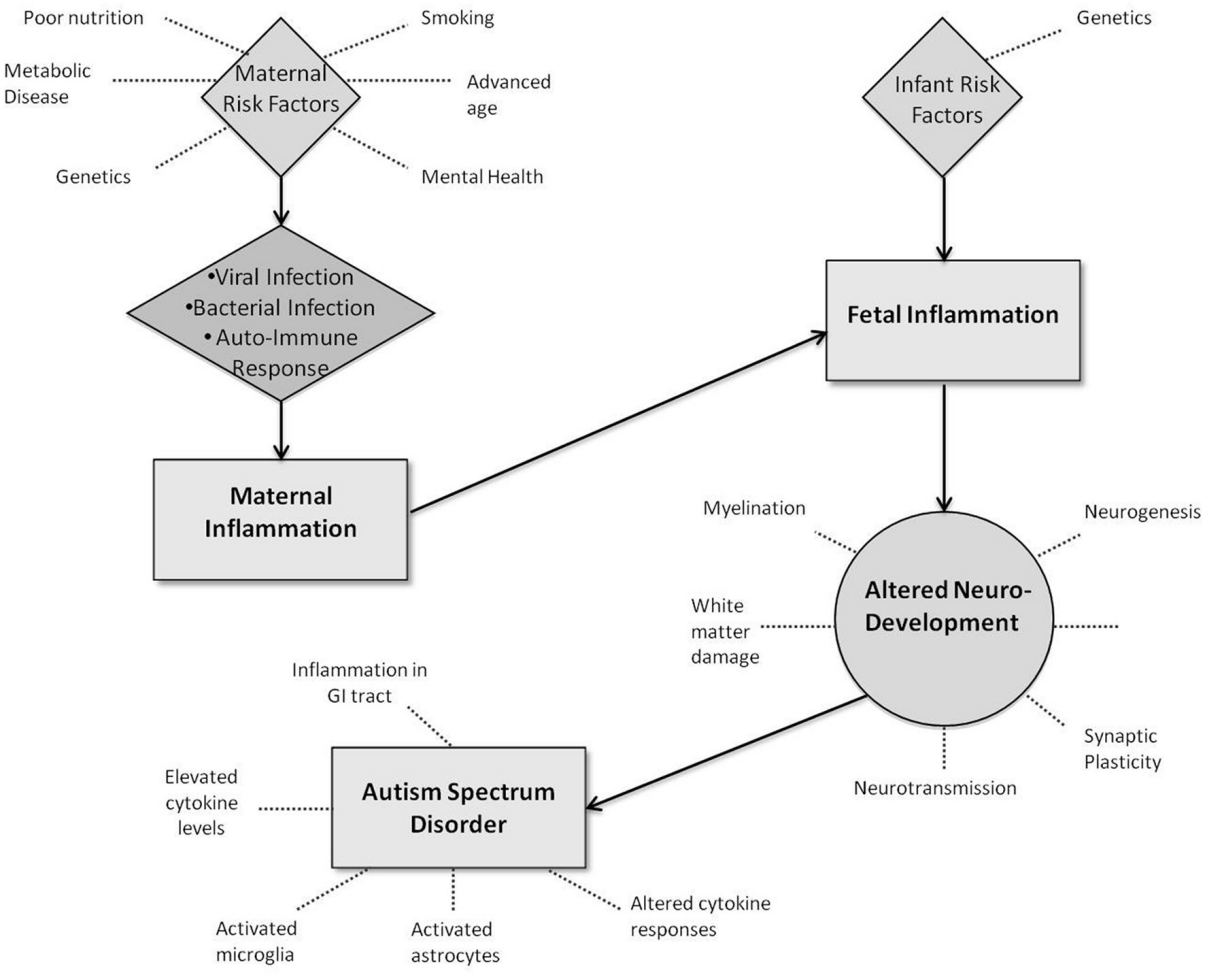
**Potential interactions between infection-led inflammation and neurodevelopment in Autism Spectrum Disorders**.

Several prospective studies of ASD in preterm population (based on parental questionnaire-based data) have demonstrated a comparatively elevated risk for ASD in preterm children, although the incidence of ASD varied widely between studies (19.4–41%) (Sargent, 1993; Zenclussen et al., 2007; Limperopoulos et al., 2008; Hack et al., 2009; Kuban et al., 2009; Moore et al., 2012). The populations examined all consisted of extremely preterm children (<28 weeks) or of very low birth weight (<1500 g), and the children were assessed for ASD at less than three years of age. Results should be interpreted with caution however, as the formal diagnosis of ASD is reached in significantly fewer individuals than implicated by positive questionnaire screening (Romero et al., 2003), and the incidence of positive screening for ASD is significantly higher for populations with other significant neurological, cognitive, and sensory sequelae (Coe and Lubach, 2005; Williams et al., 2008a).

Two prospective studies have included information on children formally diagnosed with ASD (Romero et al., 2006; Meyer et al., 2007). The first recruited 219 children (age 11 years) born before 26 weeks gestation in 1995 in the UK and Ireland and compared rates of ASD diagnosis to 152 children born at term (Meyer et al., 2007). Overall 8% of preterm children were assigned a diagnosis of ASD compared to none in the term group. Notably, 16% of preterm and 3% of term children screened positive based on a questionnaire alone, again emphasizing the need for caution when interpreting questionnaire-based screening data. Of those with a formal diagnosis, 13 had autistic disorder and three with PDD-NOS, representing a prevalence between 4 and 12 times higher than the general population (Meyer et al., 2007). The second study by Pinto-Martin and colleagues, recruited 623 infants of moderately low birth weight (<2000 g) born between 1984 and 1987 in NJ, USA (Romero et al., 2006). At age 21, the overall prevalence rate of ASD for the cohort was 5%, whereas questionnaire screening identified 18.8% as positive.

In summary, these retrospective and prospective epidemiological studies provide some evidence that prematurity, measured either as gestational age or birth weight, increases the risk and alters the symptomology of ASD. Yet it remains clear that further large prospective cohort studies of children born preterm are required to confirm the exact incidence and identify other potentially modifying risk factors. Such research also does not resolve how prematurity could mechanistically lead to the development of ASD. The answer to this question may lie with one or more of the many recognized antecedents of preterm birth, in particular, exposure to maternal/fetal infection and inflammation.

## The maternal and fetal inflammatory responses to infection

During pregnancy, maternal immunity is associated systemically with enhanced suppressor responses and greater susceptibility of the mother to a range of infections, particularly with viral and intracellular pathogens (Chheda et al., 1996). On an anatomical level, a range of active tolerance mechanisms, including the induction of regulatory T cells, hormones and anti-inflammatory cytokines, ensure that fetal rejection does not occur (Jones et al., 1996). Maternal infections can, and do result in inflammatory responses (Gotsch et al., 2007; Agrawal and Hirsch, 2012; Burd et al., 2012), and these are intimately related to the onset of spontaneous labor and preterm birth (Burd et al., 2009; Brown, 2011; Meyer et al., 2011). Additionally, ascending intrauterine bacterial infections are common among mothers who deliver preterm [up to 40% (Brown, 2012)], and this is associated with placental inflammation as well as fetal inflammatory cytokine responses (Pararas et al., 2006).

Research suggests that exposure of the developing fetus, either directly to infection and inflammation, or indirectly via maternal inflammation, can impact on development of the CNS and therefore possibly contribute to ASD. This evidence will be discussed in turn. However, a causal relationship between infection-induced inflammation and ASD has not been formally established and important mechanistic questions remain.

## What is the evidence for a role of prenatal infection in the development of ASD?

Maternal prenatal infections have been linked to neurodevelopmental and neurological disorders, including cerebral palsy (Goldenberg et al., 2000, 2008), schizophrenia and ASD (Lamont and Sawant, 2005). An association between maternal infection and schizophrenia is supported by both human studies and animal pregnancy models (Chaiworapongsa et al., 2002). Hypotheses aimed at explaining this relationship have focused on the action of inflammatory cytokines on the developing fetal brain, or the induction of deleterious maternal autoimmune responses, as likely routes to altered neurodevelopment (Lamont and Sawant, 2005). As schizophrenia and ASD share both clinical and biological links, research has subsequently turned to evaluate the role of prenatal infection/inflammation and ASD (Gavilanes et al., 2009).

### Bacterial infections

Maternal bacterial infection during pregnancy is intrinsically linked with preterm delivery (Hatfield et al., 2011). Chorioamnionitis (inflammation of the chorionic disc, maternal and/or fetal membranes, cord and/or amniotic fluid) is associated with approximately 25–40% of all preterm births (Wu, 2002; Wharton et al., 2004; Shatrov et al., 2010), and can result in elevated inflammatory markers in the amniotic fluid, cord and newborn peripheral blood and cerebrospinal fluid (Stephens et al., 2012). Alterations in brain morphology following prenatal exposure to chorioamnionitis have been reported, and include a decreased number of neurons in the cortex, hippocampus and substantia nigra (Samara et al., 2008) as well as diffuse global changes in cortical thickness (Johnson and Marlow, 2009). Exposure to clinical and histological chorioamnionitis has been associated with white matter injury and worse neurological outcomes, including cerebral palsy (Schendel et al., 2009; Johnson et al., 2010). Importantly, the presence of a fetal inflammatory response appears more predictive of brain injury than maternal inflammation (Redline et al., 1998, 2000; Yoon et al., 2000, 2001; Pinto-Martin et al., 2011).

Few studies have addressed the potential association between maternal bacterial infections and ASD. In 2010, Atladottir et al. (2010a,b) reported an association between maternal bacterial infection during the second trimester and the diagnosis of ASD in the child (adjusted hazard ration of 1.42). They were not able to discern the causative pathogens associated with this effect, but noted that the most common infections were urogenital (~75%) and suggested that the observed association could be due to “transplacental passage of maternally produced cytokines or antibodies in response to the infection” (p. 1429). Limperopoulos et al. (2008) observed that for 91 very low birth weight infants, chorioamnionitis was significantly associated with an abnormal autism screening score, with an OR of 9.669 (95% CI 3.302–28.310). In contrast to the above studies, Abdallah et al. found no significant associations between prenatal maternal infections (outpatient and hospital admissions) and the development of ASD (Abdallah et al., 2012).

Studies using animal models support the findings in humans and have identified an increased risk of several behaviors characteristic of ASD in the offspring of lipopolysaccharide (LPS)-activated mothers (Malkova et al., 2012). Such deficits include communication (ultrasonic vocalizations) (Malkova, 2010), social interaction (Smith et al., 2007), elevated anxiety and inhibition deficits (Patterson, 2009; Meyer and Feldon, 2010).

### Viral infections

There is less evidence that viral infections can increase the risk preterm birth in comparison to bacterial infections (Srinivas et al., 2006). Nevertheless, maternal viral infection is often associated with an increase in the incidence of psychiatric disorders with a neurodevelopmental origin, particularly schizophrenia (Brown and Patterson, 2011). Maternal influenza infection during the first trimester has also been associated with an increased risk of ASDs in the offspring in a study by Atladottir et al. (2010a). This finding is supported by laboratory evidence, whereby an influenza infection induced the gene expression of various proinflammatory and chemo-attractive cytokines in cultured human fetal membrane cells (Uchide et al., 2005). Furthermore, respiratory infection with the human influenza virus at mid-gestation in animal models results in behavioral and pharmacological abnormalities (Patterson, 2002; Shi et al., 2003), along with widespread reduction in gray matter volume in the cortex and reduced white matter volume in the parietal cortex (Short et al., 2010). The Atladottir and colleagues study was also subject to bias, as they were based on infections requiring admission to hospital, and did not include subclinical infections, or those treated by general practitioner.

Other viruses for which a potential association with ASD and related disorders have been suggested include congenital rubella (Freedman et al., 1970; Chess, 1971, 1977; Stubbs, 1995), herpes, cytomegalovirus (Stern and Tucker, 1973; Stubbs, 1978; Markowitz, 1983; Ivarsson et al., 1990; Ciaranello and Ciaranello, 1995; Yamashita et al., 2003; Sweeten et al., 2004), varicella (Ciaranello and Ciaranello, 1995), mumps (Ciaranello and Ciaranello, 1995), polyomavirus (Lintas et al., 2010) and enterovirus (Sadeharju et al., 2003; Johnson et al., 2009a). However, these infections are relatively uncommon and therefore evidence is restricted to case reports and hypotheses.

### Maternal autoimmune conditions

Epidemiological studies show that autoimmune diseases (rheumatoid arthritis, celiac disease, type 1 diabetes) are more common in mothers of children diagnosed with ASD than in mothers of children without developmental abnormalities (Atladottir et al., 2010b; Ashwood et al., 2011a). Animal studies have demonstrated that injections of rodents or rhesus macaques during mid-gestation with immunoglobulins isolated from human mothers of children with ASD results in abnormal stereotypic behaviors in their offspring (Martin et al., 2008; Singer et al., 2009). Furthermore, subsets of mothers of children with ASD have circulating antibodies which target fetal brain proteins [as reviewed in Wills et al. (2007)]. Immune responses to viral infections commonly result in transiently elevated levels of autoantibodies (Ludewig et al., 2004). It is therefore conceivable that increased maternal autoimmune reactivity following viral infections may effect fetal brain development, however, further studies are needed to support this hypothesis.

## How could infection-driven inflammation result in ASD? effects on brain structure and function

Intrauterine inflammation is increasingly being recognized as a key contributor to adverse neurological outcomes in infants born preterm (Goines et al., 2011). Evidence suggests that infants and children with ASD exhibit altered immunological status relative to unaffected children. For example, increased levels of pro-inflammatory cytokines (IFNγ and IL-6) were observed in the mid-gestation serum of a mother who went onto deliver a child with ASD. Further, post-mortem studies of brain and cerebrospinal fluid of individuals with ASD (8/13 by drowning) have shown an higher degree of inflammation with elevated cytokine levels and activated microglia and astrocytes compared to control subjects (Morgan et al., 2010; Lintas et al., 2012). Subjects have ranged in age, indicating that this immune-activation may begin in early life and be long-lasting (Chez et al., 2007; Morgan et al., 2010). In addition, peripheral blood mononuclear cells display altered cytokine responses to stimulation *in vitro* (Enstrom et al., 2010; Ashwood et al., 2011b; Goines and Ashwood, 2013) along with inflammation in the gastrointestinal tract of a subset of ASD children (Ashwood, 2010; Buie et al., 2010). This may be especially relevant as proinflammatory cytokines (e.g., TNFα, IFNγ, IL-1, IL-6, and IL-8) are involved in the pathogenesis of preterm infant brain injury, predominantly white matter damage (Dammann and Leviton, 1997; Yoon et al., 1997a,b; Patrick and Smith, 2002), and adversely affect neurodevelopmental processes, including neurogenesis, neuronal migration, synaptic plasticity, neurotransmission, and myelination (Zhu et al., 2002; Bauer et al., 2007; Rostene et al., 2007). Microarray studies have also shown dysregulation of several ASD candidate genes known to regulate both brain and immune system development (Careaga et al., 2010; Lintas et al., 2012).

It is difficult, however, to attribute infection-related inflammation to aberrant CNS development in individuals with ASD as ASD is a complex disorder with no common cellular, molecular or systems level unification. Yet the preterm infant may be at particular risk of neurodevelopmental disability, due to a birth occurring during the 2^nd^ trimester (23^rd^ to 27^th^ weeks) when the brain is particularly vulnerable to a heightened inflammatory state. Such a time corresponds to the transformation of oligodendrocytes, migration of neuron precursors from the germinal plate, and the up-regulation of excitatory neurotransmitter pathways. Such factors can be linked to the several neurodevelopmental anomalies noted in ASD (Shinohe et al., 2006; Hughes, 2007; Bassett and Bullmore, 2009; Wegiel et al., 2010; Deoni et al., 2011; Essa et al., 2012).

## The role of modifiers

Despite the emerging evidence for the association between maternal infection/inflammation and ASD, this relationship is not universal to all cases of ASD. This is expected, considering the large heterogeneity of ASD symptomology, and the number of risk factors currently described. It is therefore likely that specific modifying factors influence this association, effecting either protective or injurious susceptibility to ASD risk. Firstly, the interaction of infection/inflammation and ASD risk may be modulated by maternal factors during pregnancy including smoking, age, mental health and metabolic disease. And secondly, the clinical presentation among individuals may be due to gene-gene or gene-environment interaction. All modifying factors are unlikely to singularly affect susceptibility, but are likely to be inter-related and accumulative.

Several maternal factors may increase the likelihood of preterm birth including smoking during pregnancy (Simpson, 1957; Schwartz et al., 1972; Berkowitz and Papiernik, 1993; Kaminski, 1997; Shah and Bracken, 2000; Bada et al., 2005; Kyrklund-Blomberg et al., 2005; Ng and Zelikoff, 2007; McCowan et al., 2009; Thiriez et al., 2009), age greater than 35 years (Cnattingius et al., 1992; Fraser et al., 1995; Gilbert et al., 1999; Ananth et al., 2001; Jacobsson et al., 2004), metabolic syndrome (Rey and Couturier, 1994; Catov et al., 2007a,b, 2008, 2010; Edison et al., 2007; Gilbert et al., 2007; Salihu et al., 2008; Chatzi et al., 2009; Ehrenberg et al., 2009; Johnson et al., 2009b), poor nutritional status (Cogswell et al., 2003; Siega-Riz et al., 2006; Bodnar et al., 2010; Czeizel et al., 2010) and mental health (Blondel et al., 1990; Oakley et al., 1990; Bryce et al., 1991; Hedegaard et al., 1996). Several of these factors can also modulate the level of maternal inflammation during pregnancy. For example, pregnancy stress results in the section of corticotrophin-releasing hormone (CRH) from the hypothalamus, and increased plasma levels of CRH have been linked to preterm labor (Hobel et al., 1999). While some evidence suggests that such maternal risk factors can contribute to the development of ASD (Rizzo et al., 1997; Croen et al., 2002, 2007; Hultman et al., 2002; Glasson et al., 2004; Beversdorf et al., 2005; Larsson et al., 2005; Lauritsen et al., 2005; Leonard et al., 2006; Reichenberg et al., 2006; Dionne et al., 2008; Durkin et al., 2008; Grant and Soles, 2009; Grether et al., 2009; King et al., 2009; Li et al., 2009a,b; Burstyn et al., 2010; James et al., 2010; Kalkbrenner et al., 2012; Meguid et al., 2010; Roza et al., 2010; Shelton et al., 2010; Dodds et al., 2011; Lee et al., 2012; Parner et al., 2012; Rai et al., 2012; Sandin et al., 2012; Schmidt et al., 2012), results remain largely mixed and are strongest for advanced maternal age.

Variable distributions of genetic polymorphisms associated with the inflammatory response may be related to the risk of ASD development in the presence of a intrauterine infection/inflammation (Nelson et al., 2005; Wu et al., 2009). Inherited cytokine or chemokine polymorphisms influence the risk for pre and perinatal brain damage contributing to cognitive impairment (Harding et al., 2005), intraventricular hemorrhage (Adcock et al., 2003; Heep et al., 2005) and cerebral palsy (Nelson et al., 2005; Gibson et al., 2006; Wu et al., 2009). The first comprehensive gene-expression analysis of the CNS in patients with ASD (Voineagu et al., 2011), observed that immune genes and glial markers were over-expressed, supporting the findings of immune dysregulation in ASD. An epidemiological study recently observed that while siblings of children with ASD had fewer prenatal or perinatal complications than their affected siblings, such complications were significant higher than control subjects (Glasson et al., 2004). Suggesting that siblings of children with ASD have reacted differently to similar environmental stimuli, perhaps due to altered gene-environment interactions.

## Conclusions

We have summarized the extant research that preterm infants may be at increased risk of developing ASD, and how maternal infection/inflammation along with modifying gene-environment interactions may be a predisposing factor. While the underlying mechanism is not understood, the association between maternal infection/inflammation provides a promising field of enquiry. There is an evident lack of large-scale prospective studies: (i) to ascertain the true incidence of ASD among children born preterm and (ii) To characterize the risk factors preterm infants who develop ASD, specifically maternal infection/inflammation. Such future studies could also have preventative implications, where treatment to minimize such risk factors could be better implemented to minimize the risk of children born preterm developing ASD.

### Conflict of interest statement

The authors declare that the research was conducted in the absence of any commercial or financial relationships that could be construed as a potential conflict of interest.
